# Increasing Efficiency With Point-of-Care Ultrasound-Guided Arthrocentesis: A Case Report and Procedure Demonstration

**DOI:** 10.7759/cureus.62082

**Published:** 2024-06-10

**Authors:** Kristine L Schultz, Kara Johnson, Philip M Grenz, Mostafa Meleis, David P Adams, Shawn M Quinn

**Affiliations:** 1 Department of Emergency and Hospital Medicine, Lehigh Valley Health Network, University of South Florida Health Morsani College of Medicine, Bethlehem, USA

**Keywords:** demonstration, emergency department, bedside, arthrocentesis, pocus

## Abstract

Patients with chief complaints of musculoskeletal pain comprise a significant portion of emergency department (ED) visits. Identifying and utilizing methods to expedite diagnosis in these cases may help reduce ED crowding, improve outcomes, and increase patient satisfaction. We present a case in which a 52-year-old man presented to the ED with complaints of unilateral right knee pain, swelling, and stiffness. An initial plain film X-ray showed a large suprapatellar effusion over the patient’s arthritic right knee. Point-of-care ultrasound (POCUS) was used by an ED physician to facilitate a suprapatellar arthrocentesis. The patient tolerated the procedure well, remarking that he had no pain during or after its completion. POCUS can increase the accuracy, efficacy, and speed of procedures for which physicians have traditionally used landmarks or formal radiology consultations. While POCUS can prove helpful, barriers to its widespread implementation still remain. However, these barriers can be addressed with relative ease.

## Introduction

Musculoskeletal (MSK) complaints are a common emergency department (ED) presentation, estimated to account for approximately 20% of all ED visits [1]. With an aging population, the incidence of and disability from osteoarthritis is increasing, and its complications are likely to be a part of acute care complaints [2]. Soft-tissue and MSK applications of point-of-care ultrasound (POCUS) are one of the core applications of emergency ultrasound and have provided benefits in helping diagnose and treat patients in the ED with MSK disorders [3,4]. Traditionally, arthrocentesis has been performed with physical examination and anatomic landmarks alone; however, a patient’s physical examination may be limited or lack the information necessary to diagnose a joint effusion secondary to surgery or arthritis.

Dynamic ultrasound guidance during procedures is becoming more popular, and the use of ultrasound has proven superior to physical examination alone when diagnosing joint effusion [3,5,6]. The suprapatellar bursa extends approximately 6 cm superior to the patella, deep to the quadriceps tendon, and in communication with the knee joint. A joint effusion can be identified on ultrasound when there is increased hypoechoic or anechoic fluid deep in the suprapatellar recess. Additionally, more fluid tends to be drained, resulting in better patient outcomes [3,5,6]. This case report aims to demonstrate the utilization of a standard approach to a POCUS-guided knee arthrocentesis in the setting of a 52-year-old male with a swollen joint and address common barriers to POCUS utilization.

## Case presentation

**Figure 1:**
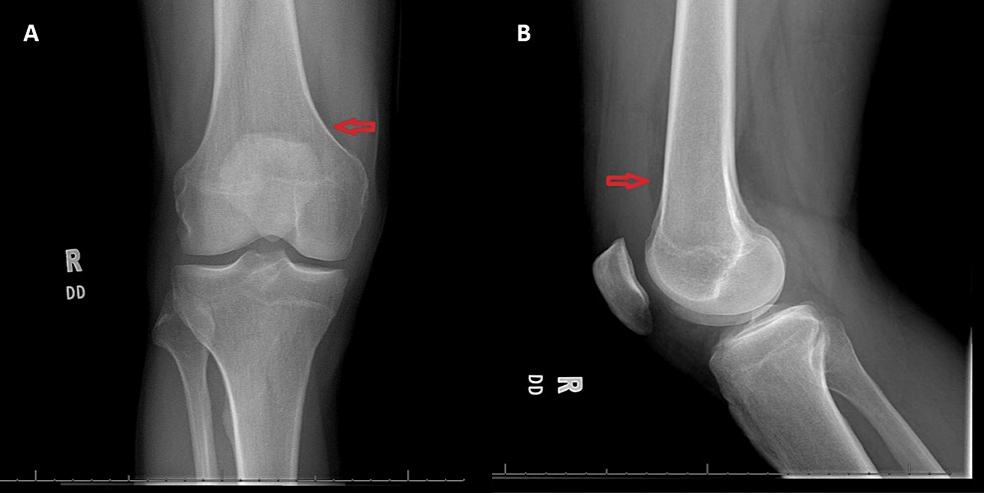
Anterior (A) and lateral (B) X-ray views of the patient’s knee featuring a suprapatellar effusion (red arrows). The findings in these images were later confirmed via POCUS. POCUS: point-of-care ultrasound

**Figure 2:**
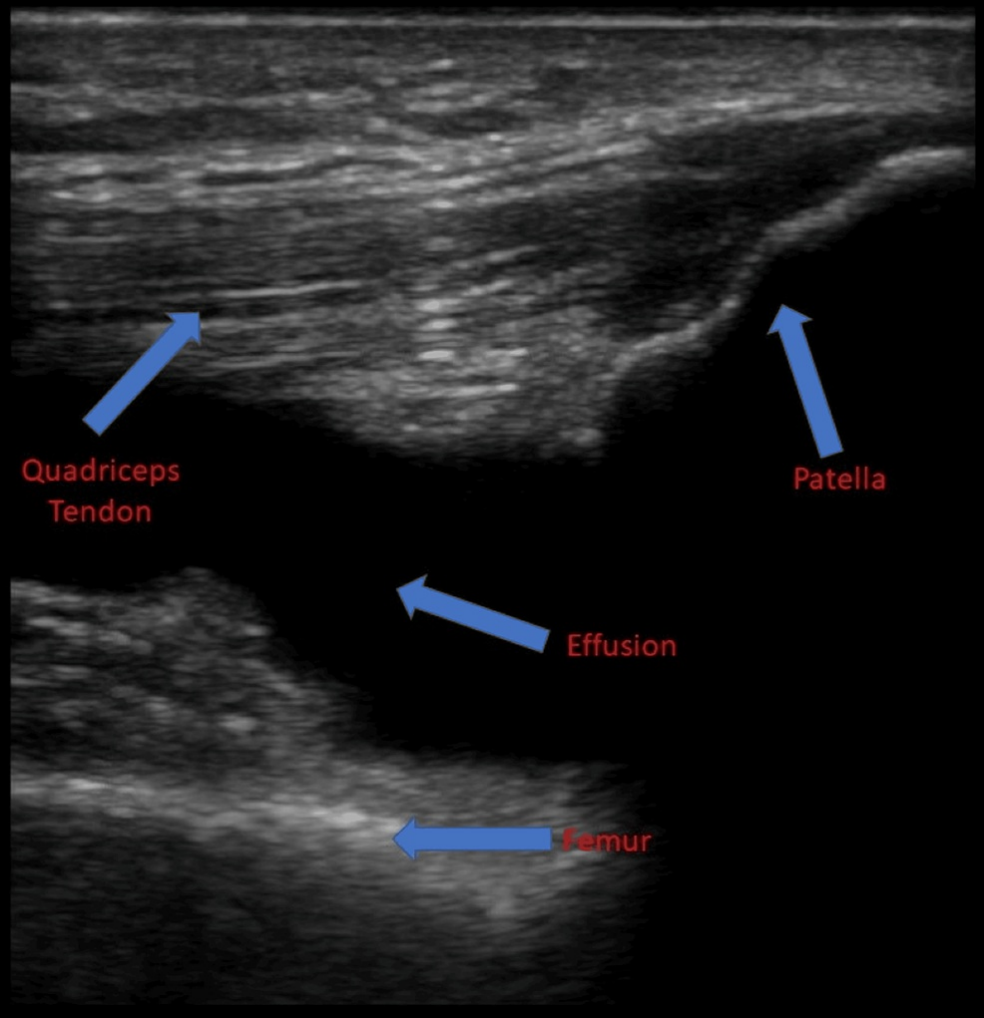
Ultrasound image using a linear probe in the longitudinal plane in the suprapatellar fossa with the indicator toward the patient's head demonstrating the effusion and anatomical structures (blue arrows).

A 52-year-old man with a past medical history of hypertension, hyperlipidemia, and osteoarthritis presented to the ED with a complaint of atraumatic right knee pain. His knee pain gradually increased over the previous week and was accompanied by swelling. Acetaminophen and ibuprofen provided minimal relief of his symptoms. On the day of the presentation, he noticed his knee was markedly swollen and stiff, which prompted him to visit the ED. He reported no history of trauma to the knee nor any twisting or “popping” sensations. The patient denied fevers, chills, or skin color changes. Aside from elevated white blood cell count (13.3 × 10^3^/μL), neutrophil count (9.9 × 10^3^/μL), and sedimentation rate (32 mm/hour), all laboratory values were normal. On examination, the patient had a moderately swollen right knee with obvious effusion and no overlying erythema or warmth. The ipsilateral ankle and hip joint were unremarkable. On arrival in the ED, the patient was given 15 mg of ketorolac intravenously, and a plain film X-ray was obtained (Figure 1). The X-ray revealed an arthritic knee with a moderate-large suprapatellar effusion. There was no fracture or dislocation. Afterward, POCUS identified the patella and femur and confirmed the suprapatellar effusion (Figure 2).

**Figure 3:**
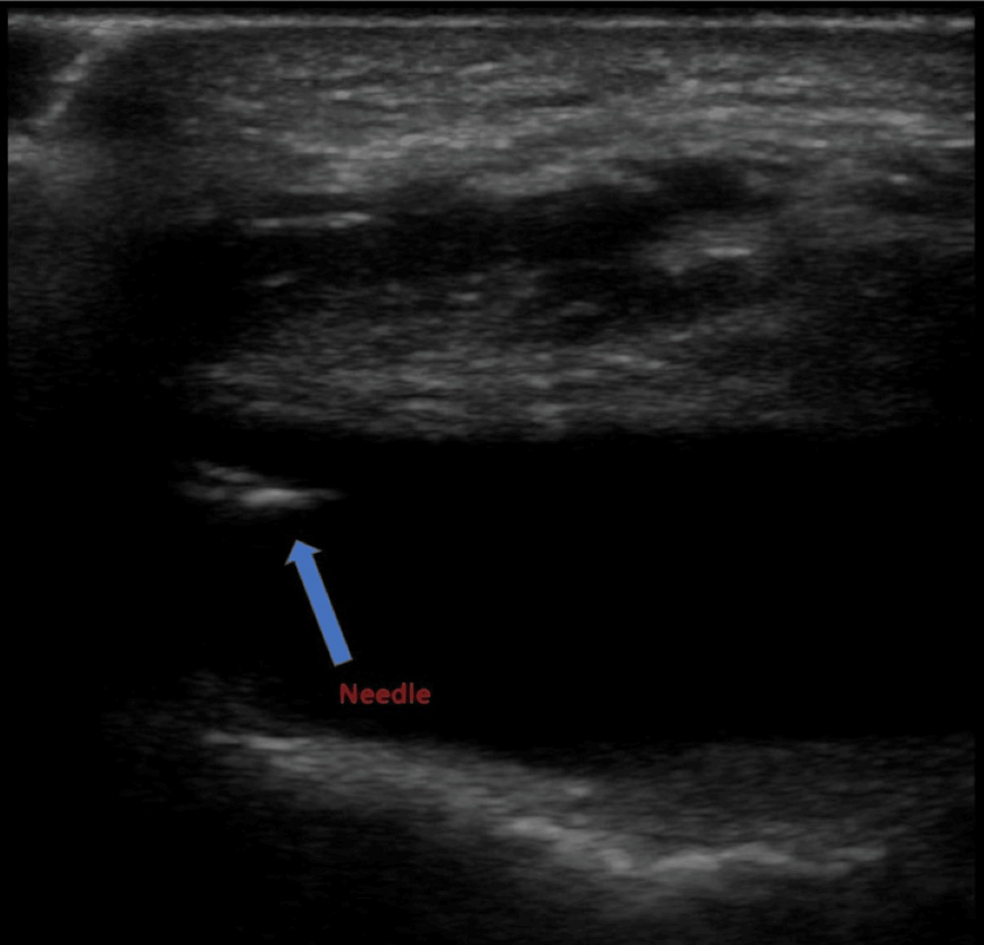
Ultrasound image in the longitudinal plane of the knee with the needle inserted into the effusion (blue arrow). This image was taken during the arthrocentesis procedure.

After a discussion of risks, benefits, and alternatives for knee arthrocentesis, the patient agreed and consented to the procedure. The patient was placed in a supine position with the head of the bed angled at 45°. His right knee was flexed at 30°, with pillows and blankets propped under the knee for support and to maintain an optimal position. The patient was prepped with a 2% chlorhexidine solution before the procedure. Approximately 5 mL of 2% lidocaine was used to anesthetize the overlying skin. An 18-gauge needle attached to a 25-mL syringe was introduced to the lateral aspect of the right knee, just superior to the patella. To visualize the effusion area, the physician placed a high-frequency linear probe in a transverse plane superior to the patella. This placement allowed visualization of both the needle and the joint effusion during the procedure (Figure 3). A sterile, water-based, bacteriostatic lubricant was used for a conducting medium, and the probe was covered by a sterile adhesive cover.

After noting these characteristics during the POCUS examination, a needle was advanced into the joint space under ultrasound guidance (Figure 3), and approximately 70 mL of yellow-tinged fluid was evacuated from the knee. The needle was removed, and pressure was placed on the injection site. POCUS was used to confirm the resolution of the effusion. The patient tolerated the procedure well and remarked that he experienced no pain during the procedure.

## Discussion

MSK complaints comprise approximately 20% of ED visits annually [1]. In a retrospective review of ED visits, 44% of patients who presented to the ED with joint-related issues complained of knee pain, and POCUS altered the course of treatment for 66% of these patients [5]. This study showed a statistically significant difference in treatment plans after POCUS was used, strongly suggesting that bedside sonography is useful in differentiating joint abnormalities and directing appropriate therapy [5]. In a second retrospective, single-center study of all ED visits for hip pain, there was a statistically significant decrease in time to bedside ultrasound vs. radiology hip ultrasound (68 vs. 208.5 minutes) and time to arthrocentesis by an emergency physician (211 minutes) versus radiology arthrocentesis (602 minutes) [7]. Given the unfavorable outcomes of a delayed diagnosis of septic arthritis, this time differential may be advantageous.

Despite the obvious benefits of performing POCUS-guided arthrocentesis, there are still some barriers to overcome, the first of which is training. While some emergency physicians may feel unqualified to use bedside ultrasound for a procedure that has traditionally been completed with the use of landmarks only, studies have shown that training sessions as short as 25 minutes significantly increased participants' self-confidence when performing this procedure [8]. Physicians may also cite time constraints, documentation requirements, and lack of equipment as potential barriers. However, Wiler et al. found no time difference between using POCUS and the standard technique and showed greater provider confidence with the procedure [9]. Supplementary demonstration videos have been included with this article, illustrating the materials (Video 1) and techniques (Video 2) necessary to perform a POCUS-assisted arthrocentesis.

**Video 1:** Listing and displaying all the materials necessary to perform an ultrasound-guided knee arthrocentesis, excluding the ultrasound machine.

**Video 2:** The authors demonstrating anatomical structures, as they appear on the ultrasound display, and proper procedure techniques.

Apart from barriers to POCUS-guided arthrocentesis, there are very few, if any, additional risks in using ultrasound guidance. While the risks of an arthrocentesis include pain, bleeding, and infection, ultrasound guidance has been proven to minimize these risks, enhancing patient satisfaction and patient outcomes [6]. As a result, joint aspirations using ultrasound guidance are safer, more effective, and faster than using the landmark technique alone [10].

## Conclusions

In this case, a 52-year-old man presented to the ED with an atraumatic moderate-large suprapatellar effusion. The patient was treated successfully with a POCUS-guided arthrocentesis and was discharged with no complications. POCUS can be used to expedite the diagnosis of joint effusions, improve confidence in the procedure, and reduce the time required to wait for radiology arthrocentesis. Aside from barriers to using POCUS, there are likely no additional risks associated with performing arthrocentesis under ultrasound guidance. Indeed, many have reported higher success rates with ultrasound versus the traditional landmark-guided approach.
